# Lightweight YOLOv8s-BD for small and dense agricultural pest detection using dynamic upsampling and bidirectional feature fusion

**DOI:** 10.3389/fpls.2026.1913220

**Published:** 2026-09-14

**Authors:** Qiang Gao, Chongchong Shi, Yu Ji, Xiaoning Chen, Huan Wang, Mengke Cao, Meili Wang

**Affiliations:** 1 Xi’an University School of Artificial Intelligence and Big Data, Xi’an, Shaanxi, China; 2 Shaanxi Joint Laboratory of Artificial Intelligence, Xi’an University, Xi’an, China; 3 School of Computer Science, Xijing University, Xi’an, China; 4 School of Information Engineering, Northwest A&F University, Yangling, China

**Keywords:** agricultural intelligence, improved yolov8s model, pest detection, real-time detection, small object detection

## Abstract

Real-time and accurate detection of crop pests in field environments is critical for intelligent plant protection and ensuring food security. However, practical farmland settings introduce substantial challenges, including small and densely distributed targets, high inter-class morphological similarity, complex backgrounds, and a long-tailed class distribution. To address these issues, an improved lightweight detection model named YOLOv8s-BD is proposed based on the YOLOv8s architecture. The Pest24 dataset is first augmented using a Generative Adversarial Network (FastGAN) to mitigate data scarcity for minority categories and enrich intra-class feature diversity. Subsequently, a dynamic upsampling module (DySample) is incorporated to enhance high-resolution feature representation for small-scale pests, and the original feature pyramid is replaced with a weighted bidirectional feature pyramid (BiFPN) to optimize multi-scale feature fusion. Experimental results demonstrate that YOLOv8s-BD achieves 72.3% mAP@0.5 and 46.9% mAP@0.5:0.95 on the Pest24 dataset, with only 7.4 million parameters and an inference speed of 1.9 ms per image, demonstrating superior accuracy and efficiency over mainstream YOLO series models. Both visualization results and error analysis confirm that the proposed model can accurately localize pest distribution regions and effectively reduce misclassification rate among similar species. The model thus offers a practical and efficient solution for multi-class pest detection in real-world agricultural scenarios.

## 1 Introduction

Crop pests are a major contributor to global yield losses. Recent advances in deep learning and IoT, driving demand for accurate pest monitoring (Kariyanna and Sowjanya, 2024) of crop pests and diseases. This creates an urgent need to develop lightweight and high-precision pest and disease detection models.

Chithambarathanu and Jeyakumar reviewed both traditional machine learning and deep learning pipelines for crop pest identification (Chithambarathanu and Jeyakumar, 2023). Their work verified that deep learning methods outperform handcrafted feature algorithms, yet detection accuracy suffers severe degradation under complex farm backgrounds, and lightweight edge deployment remains an underdeveloped research direction. Focusing specifically on deep learning pest detection frameworks, Jiang et al. further defined four widespread technical barriers: limited training samples, blurred tiny pest features, inconsistent multi-scale feature matching, and mutual occlusion of dense pests; the authors also summarized potential optimization routes for each issue (Jiang et al., 2023).

These four technical barriers widely exist in actual sticky trap detection scenes, and current YOLO-based pest detectors cannot resolve all of them simultaneously.

Although YOLO detectors strike a good balance between inference speed and recognition accuracy, they still fall short on tiny pest detection and overall robustness. Among various deep learning-based object detectors, YOLO offers unique merits for agricultural tasks: its single-stage regression framework delivers real-time inference for field equipment, achieves higher running efficiency than two-stage detectors (e.g., Faster R-CNN) and transformer models(e.g., DETR), and owns mature toolchains for edge devices. These merits make YOLOv8s our chosen baseline for targeted optimization.

Recent works have extended YOLO-based detectors for pest detection through various architectural modifications: RS-YOLO (Hou et al., 2025), a highly accurate real-time detector for small-target pests based on YOLOv8n, integrates Parallel Bottleneck, efficient multi-scale attention, and bilinear interpolation upsampling, achieving 96.6% mAP@0.5 and 81.7% mAP@0.5:0.95. DSOD-YOLO (Nie et al., 2025), a lightweight YOLOv8-based detector for small-target detection, employs a dual backbone, FASD, and CSFCN modules, and is further optimized via pruning and knowledge distillation, outperforming YOLOv9 and YOLOv10 on several small-object benchmarks. A lightweight rice pest detection model based on improved YOLOv8 (Yin et al., 2024) integrates M-CBAM attention, MPDIoU loss, and Ghost convolution, achieving 95.8% mAP with only 2.15M parameters. YOLO-LCE (Cen et al., 2025), a lightweight YOLOv8-based detector for agricultural pests, utilizes LCR modules, EPConv, Ghost modules, and WIoUv3 loss, increasing mAP50 by 1.7% while reducing parameters by 43.9% on the Pest24 dataset. A two-stage pest detection framework fusing YOLOv8 and EfficientNet within the AgriDataFusion Engine (Palanisamy and Gavaskar, 2025) achieves 91% accuracy and 89% mAP on the IP102 dataset. MSDS-YOLO (Li et al., 2026), based on YOLOv11 for small-target cotton pest detection, integrates C3k2_DMSFE, DSFPN, and NWD-CIoU loss, reaching 86.7% mAP50 on the Cottonpest2 dataset. A generalized YOLOv8-based insect detector (Vilar-Andreu et al., 2024) merges all pests into a single class, realizing universal insect detection across arbitrary crops and obtaining 96.7% mAP50. FACO-YOLO (Zheng and Jiang, 2025), an improved YOLOv11n model for underwater object detection, employs FusionFormer, ADown, CSFCN, and a lightweight detection head, increasing mAP by 1.5% and reducing parameters by 18.2%. FCBTYOLO (Yang et al., 2023), a lightweight YOLOv8n-based detector for rice pests, integrates the FCBT transformer module and uses FastGAN for data augmentation, achieving 93.6% mAP@50 with only 6.7 MB weights.

Two coupled core defects have been ignored by these single-optimization schemes: content-agnostic upsampling that fails to recover fine-grained spatial details critical for tiny targets, and inefficient multi-scale feature fusion that treats all feature levels equally, neglecting the varying importance of shallow high-resolution features for small pests versus deep semantic features for larger instances. Importantly, these two bottlenecks are not independent but mutually reinforcing-poor upsampling degrades the quality of high-resolution features fed into the fusion pyramid, while suboptimal fusion fails to deliver discriminative features to the upsampling modules, creating a vicious cycle that limits detection accuracy. To break this cycle, we propose YOLOv8s-BD built on YOLOv8s, and introduce a dual-pronged architectural improvement: the DySample dynamic upsampling module, which employs a learnable, content-aware sampling mechanism to adaptively recover boundary and texture details; and the weighted Bidirectional Feature Pyramid Network (BiFPN), which removes redundant nodes and assigns learnable per-level weights during feature fusion, automatically emphasizing the most informative scales for each pest instance. Complementing these architectural innovations, we employ FastGAN (Liu et al., 2020) to augment each of the 24 categories with 600 high-quality synthetic images. The key scientific contribution of our work lies in the synergistic integration of these three components-GAN-based augmentation enriches the training distribution for all categories, DySample ensures high-fidelity feature recovery, and BiFPN optimizes cross-scale fusion-forming a holistic solution that jointly addresses the coupled bottlenecks of small-target pest detection. Comprehensive experiments on the Pest24 dataset verify the superiority of the proposed YOLOv8s-BD model: it achieves 72.3% mAP@0.5 and 46.9% mAP@0.95 with only 7.4M parameters and 1.9 ms inference per frame, balancing high precision and lightweight inference for practical farm deployment.

## 2 Methods

### 2.1 Datasets and preprocessing

Pest24 (Wang et al., 2020) is a large-scale agricultural pest detection dataset designed for field insect monitoring in precision agriculture. The dataset comprises 24 categories of typical field crop pests, with 25,378 real-scene annotated images with more than 190,000 labeled pest instances. All images were collected from actual farmland environments using insect trapping lamps and sticky pest boards under complex field conditions involving multiple regions, crop varieties and illumination variations. Sample images of the Pest24 dataset are shown in **Figure 1**. The dataset exhibits several challenging characteristics, including extremely small target pixel scales, high inter-class morphological similarity, dense distribution and mutual occlusion of pest individuals, a severe long-tailed sample distribution, and cluttered background interference.

**Figure 1:**
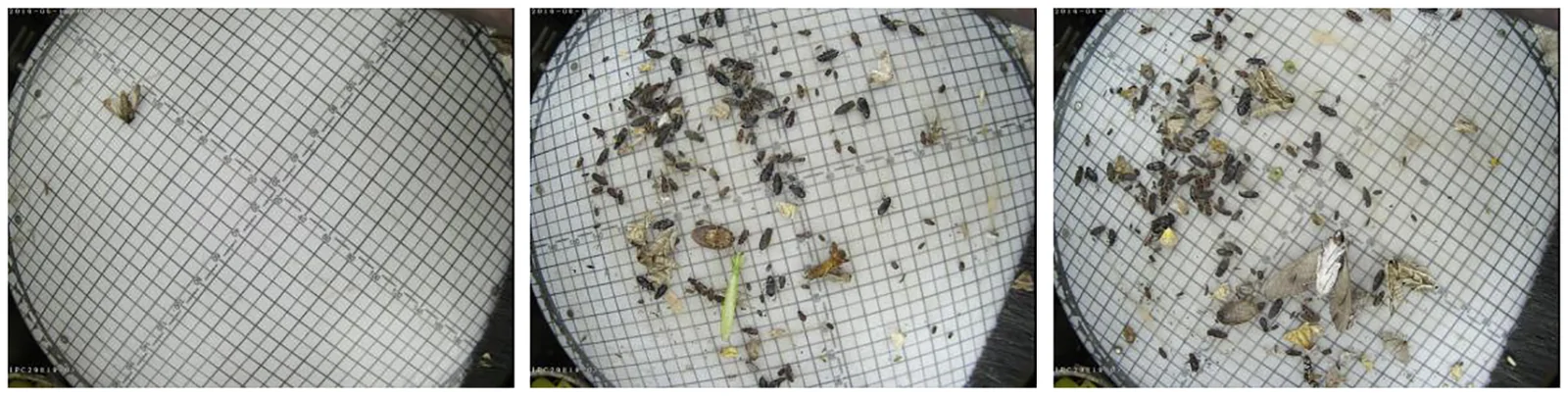
Sample images of the Pest24 dataset.

To enrich sample diversity and mitigate the overfitting risk caused by class imbalance, FastGAN (Liu et al., 2020) was adopted as a lightweight generative model for data augmentation. A large pool of synthetic images was generated for each category, followed by a rigorous two-stage quality control procedure. The generation process was quantitatively monitored using the Fréchet Inception Distance (FID) score, and the checkpoint with the lowest FID for inference. Manual visual inspection was then conducted by randomly sampling generated images from each category. Three researchers with entomology backgrounds independently evaluated these samples based on morphological integrity, background naturalness, and species recognizability using a 1–5 Likert scale. Images with an average score below 3.5 or with obvious artifacts were discarded. After this quality filtering, 600 high-quality synthetic images were retained for each category, yielding a total of 14,400 additional samples (24 × 600) as illustrated in **Figure 2**. These synthetic images were then merged with the original real training images to form the final augmented training set. For each category, the final image count thus corresponds to the sum of its original real samples and 600 generated samples, as presented in **Table 1** (e.g., the Holotrichia Oblita category comprises 90 real and 600 synthetic images, totaling 690 images). To prevent any data leakage, all FastGAN training and synthetic image generation were strictly confined to the official training split of the Pest24 dataset. The test set was completely isolated throughout the entire process and was not exposed to GAN training or synthetic data generation, thereby ensuring unbiased evaluation of the subsequent detection model.

**Figure 2:**
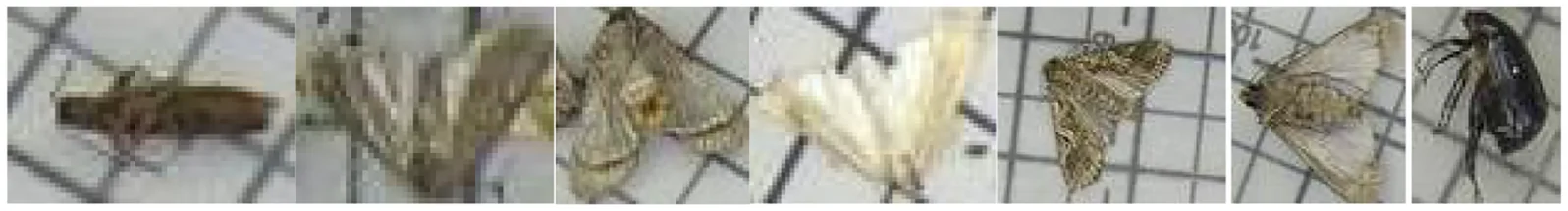
Illustration of the FastGAN-based pest sample synthesis strategy.

**Table 1:** Sample statistics of Pest24 data set category after GAN enhancement.

Index	Pest type	Number of images	Number of labels	Index	Pest type	Number of images	Number of labels
1	Bollworm	9134	28102	13	Plutella Xylostella	1131	1553
2	Meadow Borer	5551	16541	14	Holotrichia Parallela	3711	12275
3	Gryllotalpa Orientalis	3982	6974	15	Rice Planthopper	916	2111
4	Little Gecko	2524	4300	16	Yellow Tiger	1988	2286
5	Agriotes Fuscicollis Miwa	1815	6485	17	Land Tiger	959	1075
6	Nematode Trench	718	767	18	Eight-character Tiger	754	768
7	Athetis Lepigone	8120	30939	19	Holotrichia Oblita	690	708
8	Scotogramma Trifolii Rottemberg	3823	5279	20	Stem Borer	1957	2404
9	Armyworm	4428	9480	21	Striped Rice Bore	1054	1885
10	Spodoptera Cabbage	2307	2904	22	Rice Leaf Roller	1544	1840
11	Anomala Corpulenta	5828	53947	23	Spodoptera Litura	2488	2551
12	Spodoptera Exigua	4214	7863	24	Melahotus	839	1368

The manual inspection criteria were specifically designed to prioritize detection-relevant attributes. Morphological integrity ensures that the generated pests preserve recognizable species-specific shape and texture features necessary for accurate classification; background naturalness guarantees that the synthesized scenes mimic realistic field environments, enabling the detector to learn domain-relevant contextual cues; and species recognizability confirms that the generated images contain clearly identifiable pest instances that can serve as effective training samples for bounding box regression and classification. The generated images were intended to complement rather than replace real data, preserving the authentic data distribution while expanding its support in the feature space.

To mitigate the risk of introducing bias or misrepresenting real-world conditions, three design principles were followed. First, all FastGAN training and synthetic image generation were confined to the official training split, with the test set kept completely separate throughout. Second, a conservative augmentation strategy was adopted: instead of oversampling minority classes to full balance-which would artificially inflate their representation and distort the real-world class prior-a fixed number of 600 synthetic samples was added per category. This approach alleviates extreme data scarcity for tail classes without fundamentally altering the natural long-tailed distribution. Third, the manual quality filtering threshold (average score ≥ 3.5) serves as a safeguard against unrealistic artifacts that could otherwise mislead the detector into learning spurious features. Together, these measures ensure that the augmented training set remains faithful to real-world conditions while providing sufficient diversity to improve model robustness, particularly for under-represented pest categories.

**Table 1** gives the category numbers, species names, the number of various sample images and the number of labeling target boxes of 24 kinds of pests in Pest24 data set expanded by FastGAN, which fully shows the category distribution and sample size of the data set. **Figure 3** visualizes the comparison of image and label counts between the original and GAN-augmented datasets.

**Figure 3:**
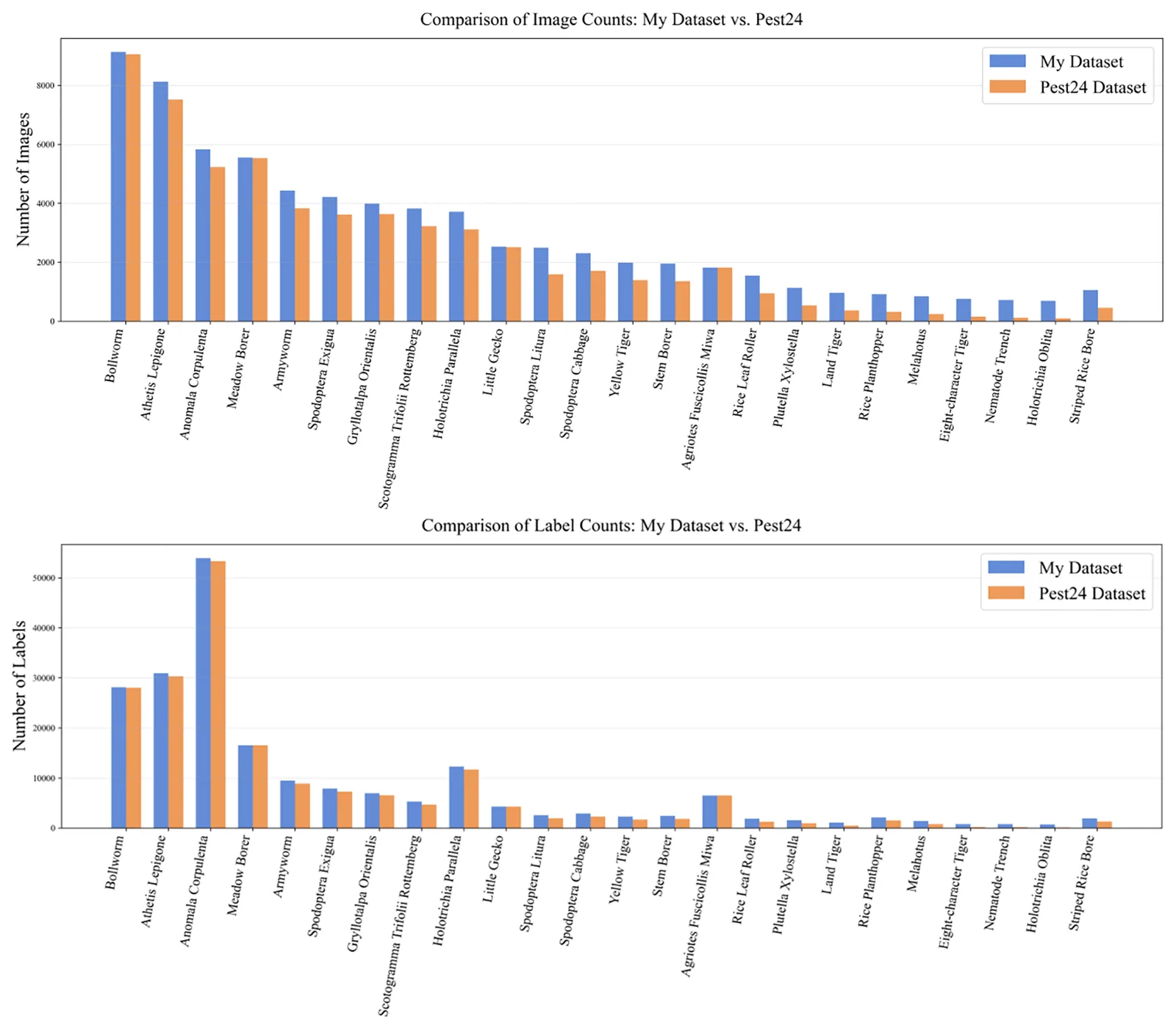
Comparison of image and label counts between the original pest24 dataset and the GAN-augmented dataset.

### 2.2 Small object detection model based on improved YOLOv8s-BD

To address insufficient feature representation and low detection accuracy of small objects in complex scenes, this paper presents an improved model named YOLOv8s-BD, as depicted in **Figure 4**. Following the basic structure of YOLOv8s, the proposed model retains the original backbone for hierarchical feature extraction and the detection head for target prediction, and the neck network is primarily optimized for multi-scale feature fusion. The original PAN-FPN structure is replaced with a Bidirectional Feature Pyramid Network (BiFPN) and DySample modules to enable bidirectional weighted feature fusion and learnable upsampling. This design effectively integrates shallow spatial details with deep semantic information, mitigating the issue of feature dilution for small objects during downsampling. It significantly improves the detection performance (Lin et al., 2017) of small objects while maintaining favorable computational efficiency to meet practical application requirements.

**Figure 4:**
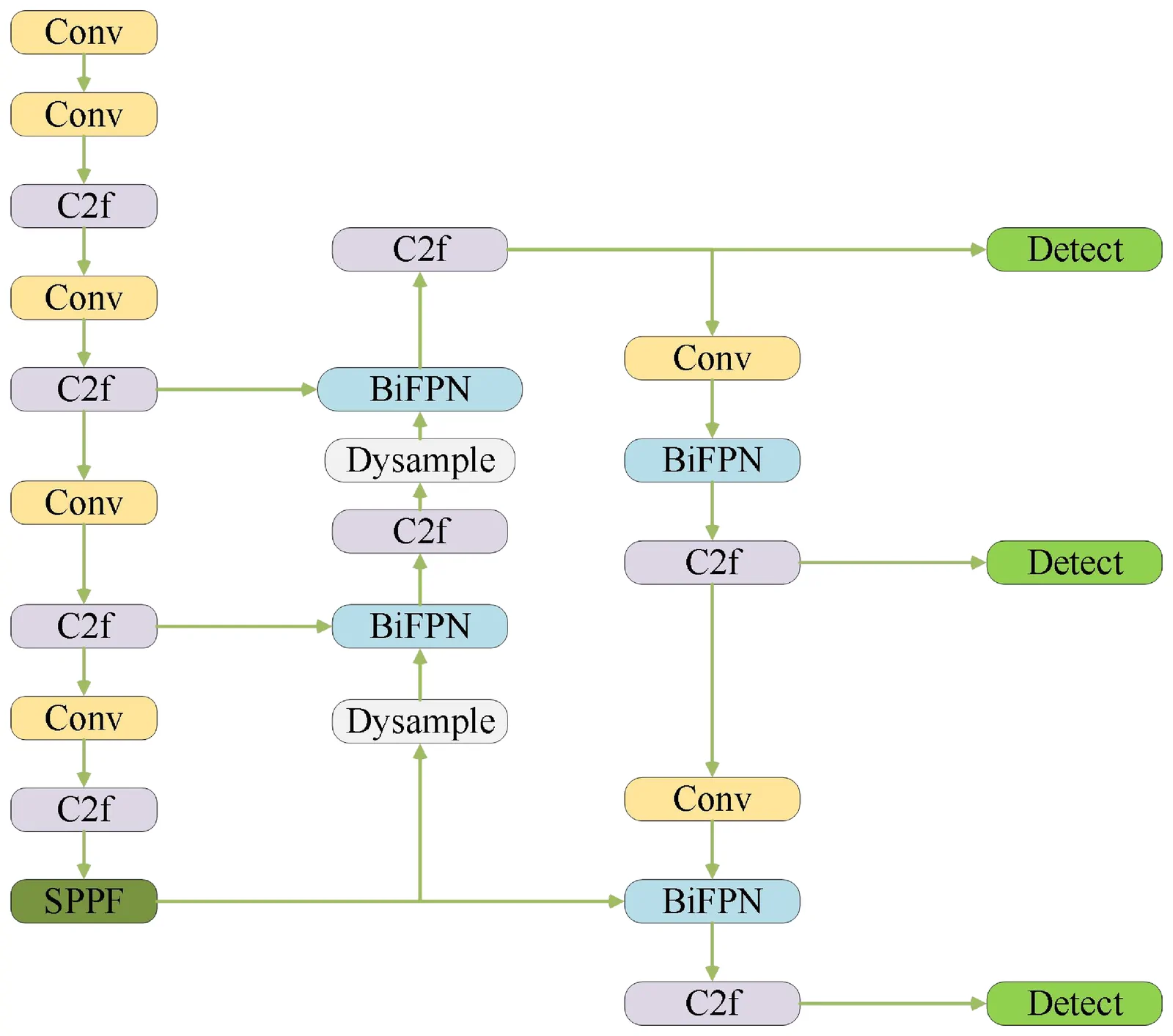
Structure of YOLOv8s-BD network.

### 2.3 Dysample module

The proposed Dysample (Liu et al., 2023) module is designed as a lightweight, multi-mode upsampling block to enhance the representation capability of high-resolution features while maintaining computational efficiency. As illustrated in **Figure 5**, DySample supports two operational modes, namely Static Scope Factor and Dynamic Scope Factor, both of which aim to adaptively aggregate context information from different receptive fields prior to pixel-wise upsampling.

**Figure 5:**
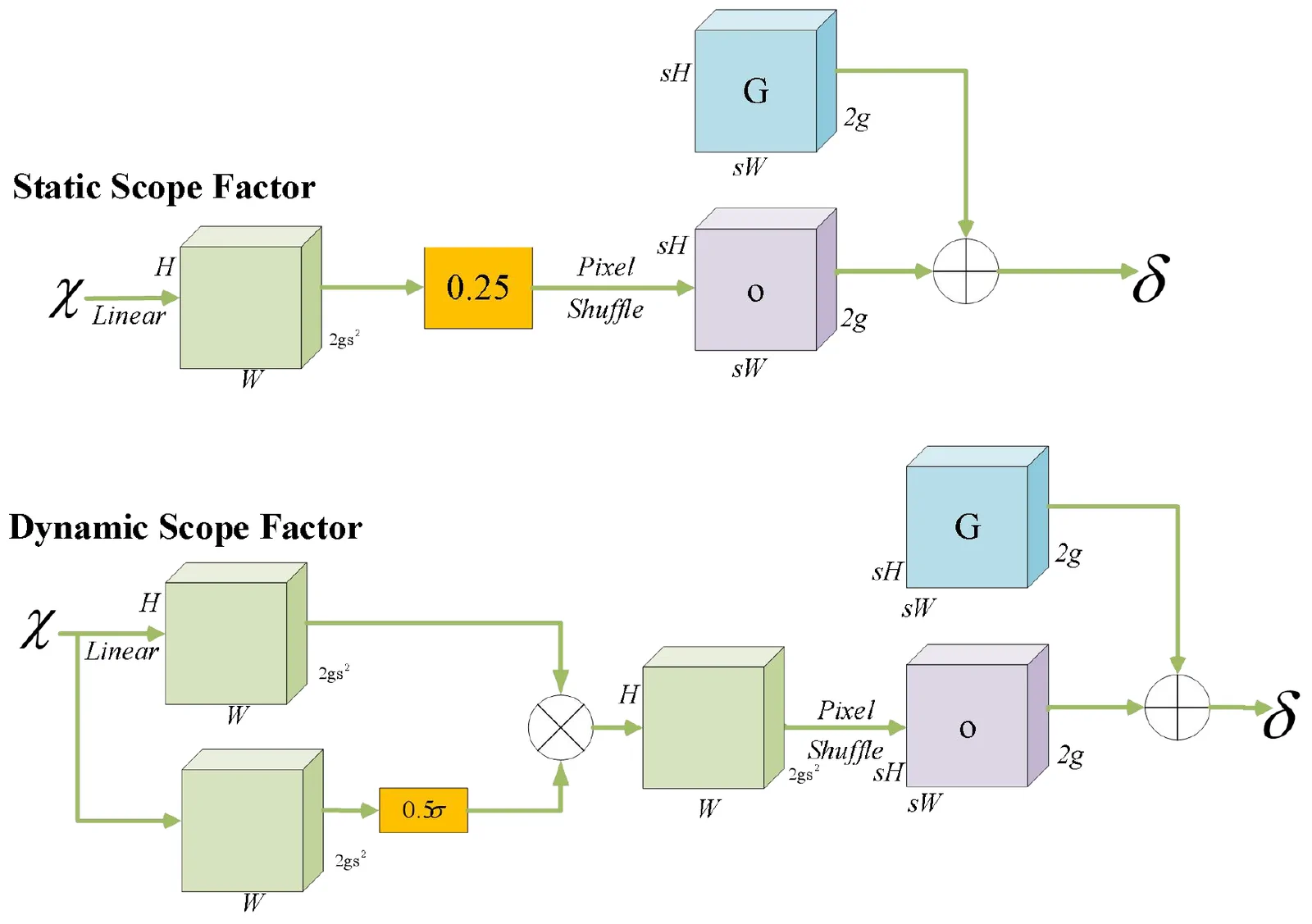
Static and dynamic scope branches in the Dysample module.

In the Static Scope Factor mode, the input feature χ is first linearly projected to a higher-dimensional space with *2gs^2^* channels, where *g* denotes the number of groups and *s* is the upsampling scale. The feature is then downscaled by a fixed factor of *0.25* to reduce spatial resolution, followed by pixel shuffle to recover the target resolution (*sH, sW*). The processed feature is subsequently combined with a context feature *G* through element-wise addition to produce the final output *δ*. This mode provides a simple yet effective means of aggregating contextual information where object scales remain a predefined scope, suitable for scenarios with relatively stable.

In the Dynamic Scope Factor mode, a learnable mechanism is introduced to adjust the aggregation scope adaptively. The input feature χ is split into two branches: one branch performs linear projection as in the static mode, while the other applies a learnable factor *0.5σ* to modulate the feature before multiplication with the main branch. This modulated feature is then processed by a second linear projection, followed by pixel shuffle to restore resolution. Finally, the output is fused with the context feature G via element-wise addition to obtain *δ*. The dynamic mode allows the model to adjust the context aggregation scope according to the input content, thereby improving robustness to variations in object scale and background complexity a property that is particularly beneficial for detecting tiny and multi-scale pests in the Pest24 dataset.

By integrating both static and dynamic modes, the DySample module offers flexible and efficient upsampling with adaptive context aggregation, effectively improving the representation of small objects without introducing excessive computational overhead.

### 2.4 Weighted bidirectional feature pyramid

Standard feature pyramid networks (FPN) (Gong et al., 2021) propagate semantic information solely in a top-down manner and thus fail to incorporate shallow detail features back into deeper layers. The path aggregation network (PANet) (Liu et al., 2018) introduces an additional bottom-up pathway to address this issue (**Figure 6**). Nevertheless, both FPN and PANet rely on simple addition or concatenation for feature fusion, treating all feature layers equally and disregarding their varying importance. Moreover, PANet contains redundant nodes with only one input edge, leading to unnecessary computational overhead without meaningful feature interactions.

**Figure 6:**
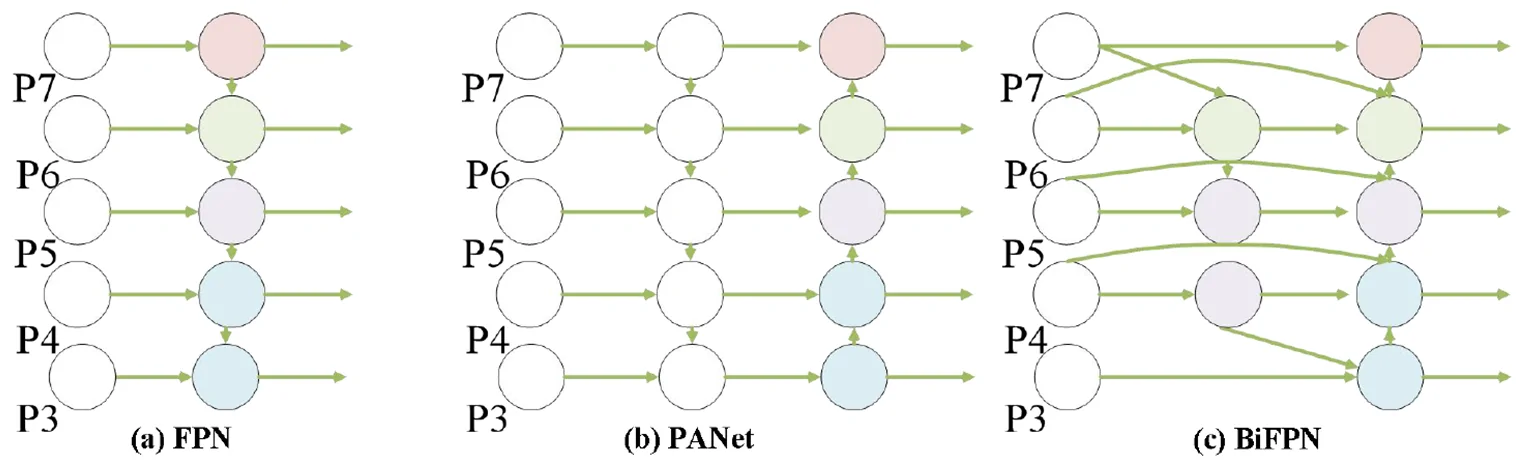
Schematic diagrams of three feature pyramid networks: **(a)** FPN, **(b)** PANet, and **(c)** BiFPN.

To overcome these limitations, we introduce the bidirectional feature pyramid network (BiFPN) (Tan et al., 2020) as our multi-scale feature fusion module. Originally proposed by Tan et al. in EfficientDet, BiFPN incorporates two key innovations. First, it removes nodes that have only one input edge, simplifying the network topology while preserving essential multi-scale feature interactions. Second, BiFPN employs weighted feature fusion, where each input feature map is assigned a learnable weight:


$$\text{O}=\frac{\sum_{\text{i}}{\text{W}}_{\text{i}}{\text{F}}_{\text{i}}}{\epsilon +\sum_{\text{j}}{\text{W}}_{\text{j}}}$$

This mechanism enables the network to adaptively emphasize informative feature levels while suppressing less relevant ones.

The advantages of BiFPN are particularly valuable for the Pest24 dataset, which contains 24 pest categories with substantial scale variations and numerous small targets. The weighted fusion mechanism naturally assigns higher weights to high-resolution shallow features when detecting small pests, while emphasizing deep semantic features for larger instances. In addition, the bidirectional information flow preserves fine-grained texture details that are critical for distinguishing visually similar pest species. These characteristics make BiFPN a suitable choice for pest detection in agricultural settings.

### 2.5 Environmental configuration and parameter settings

In this study, the Pest24 dataset was randomly divided into training, validation, and test sets with a ratio of 6:2:2 according to the number of images. All experiments were conducted on a computing server equipped with an NVIDIA A40 48GB GPU running the Ubuntu 20.04.6 LTS operating system. The software environment was configured with Python 3.11, CUDA 12.1, PyTorch 2.1.0, and Torchvision 0.16.0. The training hyperparameters were set as follows: batch size 32, epoch 300, initial learning rate 0.01, weight decay 0.0005, optimizer SGD, and input image size 640×640 pixels. To ensure a fair and unbiased comparison with baseline models, all hyperparameters were taken directly from the official YOLOv8 defaults without any dataset-specific tuning or optimization. This fixed-configuration protocol ensures that any performance improvements can be attributed solely to the architectural innovations introduced in this work.

For data preprocessing, the following steps were applied to all input images following the standard YOLOv8 training pipeline. During training, online data augmentation was employed to enhance model generalization, including Mosaic augmentation (combining four training images, probability 1.0), random horizontal flipping (probability 0.5), random scaling (scale range 0.5), and HSV color-space augmentation (hue ±0.015, saturation ±0.7, value ±0.4). All input images were resized to 640×640 pixels and pixel values were normalized to the [0, 1] range by dividing by 255. These augmentations were disabled during validation and testing to ensure unbiased evaluation. The same preprocessing pipeline was applied consistently to all compared models. The proposed YOLOv8s-BD is subsequently compared against several mainstream YOLO series models (YOLOv5, YOLOv7, YOLOv8, YOLOv10, and YOLOv11), with detailed comparison results reported in Section 3.1.

### 2.6 Evaluation index

For agricultural pest detection, evaluation metrics are selected following conventional standards in agricultural machine vision and object detection, with consideration given to accurate identification, full category coverage, and precise localization in complex field environments.

Precision (Zhen et al., 2025), Recal (Peng et al., 2023), mAP@0.5 (Shi et al., 2025), and mAP@0.5:0.95 are used as the main evaluation indicators. The basic concepts of detection outcomes are first introduced as follows: TP (True Positive) refers to the number of pest instances correctly detected by the model, FP (False Positive) denotes the number of non-pest objects or background regions incorrectly detected as pests, and FN (False Negative) indicates the number of pest instances that are present but are missed by the model.

Based on these definitions, Precision (P) is calculated as the ratio of correctly detected pests to all predicted pest samples, which reflects the model’s ability to suppress false positives:


$$\text{Precision}=\frac{\text{TP}}{\text{TP}+\text{FP}}$$

Recall (R) represents the proportion of actual pest samples that are correctly detected by the model. This metric characterizes the model’s ability to reduce the omission of real pest targets, and its computational formula is given as follows:


$$\text{Recall}=\frac{\text{TP}}{\text{TP}+\text{FN}}$$

The F1 score is relevant for evaluating both accurate identification and comprehensive coverage in disease detection. The calculation formula is as follows:


$$\text{F}1=2\times \frac{\text{Precision}\times \text{Recall}}{\text{Precision}+\text{Recall}}$$

MAP (mean average precision) is one of the core indicators in target detection and is divided into the following common variants: mAP@0.5: the average AP (Average Precision) across all growth stage categories at an IoU threshold of 0.5.


$$\text{mAP}=\frac{\sum_{\text{i}=1}^{\text{C}}{\text{AP}}_{\text{i}}}{\text{C}}$$

mAP@0.5:0.95 refers to the mean Average Precision calculated at multiple Intersection over Union (IoU) thresholds, ranging from 0.5 to 0.95 in increments of 0.05. At each threshold, the mAP is computed and subsequently averaged, providing a comprehensive evaluation of model performance across varying localization accuracy criteria.


$$\text{mAP}@0.5:0.95=\frac{\sum_{\text{t}=0.5}^{0.95}\text{mAP}@t}{10}$$

For disease identification, mAP@0.5 measures the ability to roughly position the growth stage, and mAP@0.5 0.95 evaluates the ability to accurately frame and position the growth stage.

To verify that the observed performance improvements are statistically reliable, independent-sample t-tests (for comparative experiments) and paired-sample t-tests (for ablation experiments) were conducted on the core metrics (mAP0.5, mAP0.5-0.95). For the proposed YOLOv8s-BD, we performed 5 independent training runs with different random seeds to assess model stability. For the ablation experiments, each configuration was independently trained 5 times with fixed random seeds and consistent hardware environments. Using the 5 repeated runs as samples, a significance level of α=0.05 was adopted: a *p*-value < 0.05 considered statistically significant.

## 3 Results

### 3.1 Performance between YOLOv8s-BD and other models

To comprehensively evaluate the effectiveness of the proposed YOLOv8s-BD, a comparison was conducted with several state-of-the-art YOLO series models on the Pest24 dataset, including YOLOv5, YOLOv7, YOLOv8, YOLOv10, and YOLOv11. The comparison metrics include precision (P), recall (R), mAP@0.5, mAP@0.5:0.95, number of parameters, GFLOPs, and inference speed, as shown in **Table 2**.

**Table 2:** Comparative experiment.

Model	P/%	R/%	mAP0.5/%	mAP0.5–0.95/%	Parameters/×10^6^ M	GFLOPs	Inference speed (ms)
YOLOv5	74.1	66.0	68.8	42.6	7.1	16.0	1.5
YOLOv7	76.5	67.7	71.3	45.2	36.6	103.6	2.8
YOLOv8	73.3	67.4	70.6	45.3	11.2	28.5	2.5
YOLOv10	68.7	70.1	71.4	45.7	7.2	21.5	2.1
YOLOv11	72.8	65.1	70.1	45.0	9.42	21.4	2.4
YOLOv8s-BD	73.1	69.2	72.3	46.9	7.4	25.1	1.9

The experimental settings are kept consistent for all models to ensure the fairness and comparability of the performance comparison results. For YOLOv8s-BD, results are reported as mean values from 5 independent training runs with different random seeds. Compared models were evaluated under identical test conditions using their official pre-trained weights. The standard deviations for mAP@0.5 across all configurations ranged from 0.13% to 0.20%, confirming the stability of the results. Bold values denote metrics of the final selected YOLOv8s‑BD model.

Among all compared models, YOLOv8s-BD achieves the highest overall detection performance Its mAP@0.5 reached 72.3%, which is 3.5%, 1.0%, 1.7%, 0.9%, and 2.2% higher than YOLOv5 (Jocher, 2020), YOLOv7 (Wang et al., 2023), YOLOv8 (Sohan et al., 2024), YOLOv10 (Wang A. et al., 2024), and YOLOv11 (Khanam and Hussain, 2024), respectively. The mAP@0.5–0.95 of YOLOv8s-BD was 46.9%, outperforming all other models and indicating superior multi-scale detection capability, particularly for small and densely distributed pests in the Pest24 dataset. In terms of model complexity,YOLOv8s-BD maintains a lightweight design with only 7.4M parameters and 25.1 GFLOPs, substantially lower than YOLOv7 (36.6M, 103.6 GFLOPs) and comparable to YOLOv5 and YOLOv10.The inference speed is 1.9 ms per image, faster than YOLOv8 (2.5 ms), YOLOv11 (2.4 ms), and YOLOv7 (2.8 ms), demonstrating indicating that the proposed architectural modifications do not compromise inference efficiency.

Overall, YOLOv8s-BD achieves a more favorable balance between detection accuracy and computational cost than other YOLO series models, making it better suited for practical agricultural pest detection scenarios with limited computing resources.

Independent-sample analysis using independent-sample *t*-tests confirms that YOLOv8s-BD achieves significantly higher detection accuracy compared with all other YOLO variants (*p* < 0.05 for all comparisons). The standard deviations across 5 repeated runs were consistently low, confirming the stability of the results. These findings indicate that the observed performance improvements are statistically robust and not attributable to random variation.

### 3.2 Ablation experiments

To validate the effectiveness of each proposed module, ablation experiments were conducted on the GAN-augmented dataset using YOLOv8s as the baseline model. The results are summarized in **Table 3**.

**Table 3:** Experimental results of YOLOv8s-BD model ablation.

CSFCN	Bifpn	Dysample	P/%	R/%	mAP0.5/%	mAP0.5_–0.95_/%	Parameters/×10^6^ M	GFLOPs	Inference speed(ms)
×	×	×	73.3	67.4	70.6	45.3	11.2	28.5	2.5
√	×	×	75.8	68.2	71.3	45.8	12.5	31.6	2.3
×	√	×	70.2	70.9	71.8	46.5	7.4	25.0	2.1
×	×	√	70.8	69.7	71.3	45.8	11.1	28.5	2.0
√	√	×	70.8	69.1	71.7	46.1	7.6	27.3	2.5
**×**	**√**	**√**	73.1**	69.2**	72.3**	46.9**	**7.4**	**25.1**	**1.9**
√	×	√	69.0	70.7	71.8	46.3	12.5	31.6	2.2
√	√	√	74.5	70.0	72.0	46.0	7.6	27.3	2.2

“√” indicates the adoption of the corresponding strategy, “×” indicates the nonadoption of the corresponding strategy. All models in this table are trained on the GAN-augmented dataset. **indicates extremely significant difference compared with the baseline (*p* < 0.01) based on paired-sample t-tests. Each ablation experiment was repeated 5 times, and the average value was taken as the final result. Standard deviations for mAP@0.5 ranged from 0.13% to 0.20% across all configurations, confirming the stability of the results.

The baseline YOLOv8s achieved a P of 73.3%, R of 67.4%, mAP@0.5 of 70.6%, and mAP@0.5:0.95 of 45.3%, with 11.2×10^6^ parameters, 28.5 GFLOPs, and an inference speed of 2.5 ms per image.

When only the BiFPN module was incorporated the parameter count was reduced to 7.4×10^6^ and GFLOPs to 25.0, while inference speed improved to 2.1 ms. Meanwhile, mAP0.5 and mAP0.5-0.95 increased to 71.8% and 46.5%, respectively. These results indicate that the lightweight bidirectional feature fusion structure of BiFPN effectively reduces model complexity while enhancing multi-scale feature representation. When only the DySample module was introduced, the parameter count and GFLOPs remained nearly identical to the baseline, yet inference speed improved to 2.0 ms and mAP0.5 to 71.3%. This confirms that the dynamic upsampling strategy optimizes feature reconstruction quality without incurring additional computational overhead, yielding a moderate improvement in detection accuracy.

When BiFPN and DySample were integrated (corresponding to the sixth row in **Table 3**), the model retained 7.4×10^6^ parameters and 25.1 GFLOPs, with inference speed further improved to 1.9 ms. This combination achieved the highest performance among all configurations, with mAP0.5 reaching 72.3% and mAP0.5-0.95 reaching 46.9%.

Paired-sample t-test confirmed the statistical significance of these improvements. For the full model (BiFPN + DySample), the mean mAP@0.5 across 5 independent runs was 72.3% with a standard deviation of 0.13%, demonstrating stable and reproducible performance. Compared with the baseline YOLOv8s (70.6% ± 0.20%), the improvement of 1.7% was statistically significant (*t* = 4.98, *p* = 0.008 < 0.01), with a 95% confidence interval of 1.2–2.2%. The improvement over BiFPN alone (71.8% ± 0.15%) was 0.5% (*t* = 3.21, *p* = 0.038 < 0.05; 95% CI: 0.3–0.7%), while the improvement over DySample alone (71.3% ± 0.17%) was 1.0% (*t* = 4.12, *p* = 0.014 < 0.05; 95% CI: 0.7–1.3%). These results confirm that the combination of BiFPN and DySample achieves a statistically significant and synergistic performance gain over each individual module.

The integration of the CSFCN module was also explored to further enhance feature extraction. Experimental results indicated that while CSFCN alone slightly improved detection accuracy, it substantially increased the parameter count (by up to 12.5×10^6^) and computational cost. When combined with BiFPN, CSFCN did not provide consistent performance improvements and instead reduced inference efficiency. For example, the three-module combination (CSFCN+BiFPN+Dysample) achieved a lower mAP@0.5 of 72.0% and a slower inference speed of 2.2 ms, which is contrary to the primary design objective of a lightweight and efficient detector.

Considering the trade-off between detection accuracy, model size, and inference speed, the combination of BiFPN and DySample was selected as the core enhancement for YOLOv8s-BD. This configuration achieves an optimal balance between performance and efficiency.

### 3.3 Test experiments

To visually verify the effectiveness of our proposed YOLOv8s-BD model, we conducted a qualitative comparison against several mainstream YOLO variants (YOLOv5, YOLOv7, YOLOv8, YOLOv10, and YOLOv11) on the agricultural pest dataset. **Figure 7** presents the detection results on representative test images.

**Figure 7:**
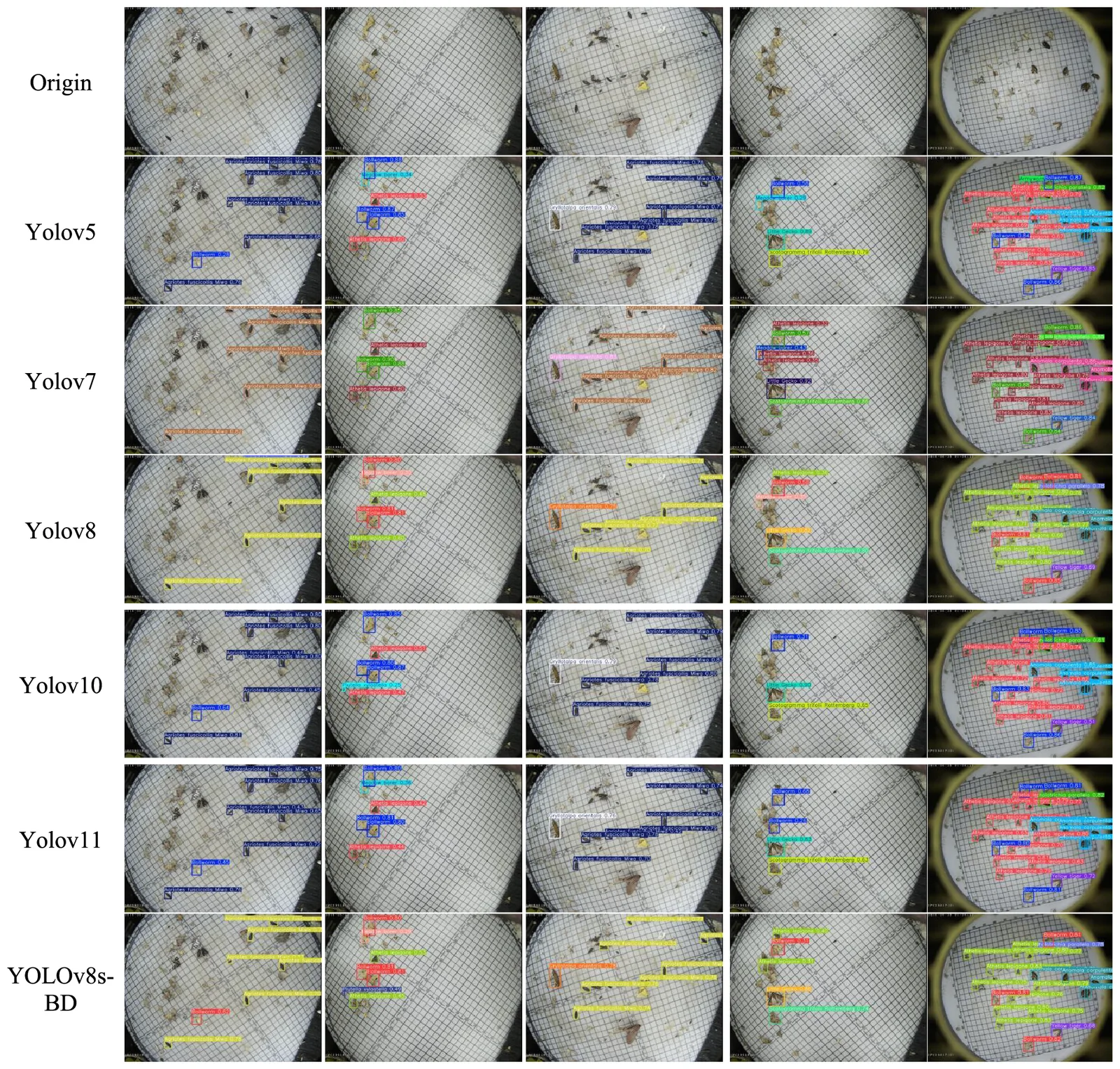
Visual pest detection results of different YOLO models.

As shown in the figure, the original images contain numerous small, densely distributed pests against complex backgrounds, posing significant detection challenges. The comparison reveals clear performance differences across models. YOLOv5 and YOLOv10 suffer from severe missed detections, failing to identify many small pests and producing incomplete or misaligned bounding boxes in dense scenes. YOLOv7 exhibits partial detection capability but struggles with overlapping and low-contrast targets, resulting in a high false negative rate. YOLOv8 and YOLOv11 show improved performance, with fewer missed detections than earlier models, though they still occasionally fail to capture fine-grained features of tiny pests, particularly in cluttered backgrounds.

In contrast, the proposed YOLOv8s-BD model demonstrates the most robust and accurate detection performance across all test cases. Benefiting from the bidirectional cross-scale connections and weighted feature fusion of the BiFPN neck, combined with learnable DySample upsampling modules that adaptively recover fine-grained spatial details, the model effectively captures both high-level semantic information and low-level spatial features. It successfully identifies almost all small, occluded, and densely distributed pests, providing complete and precise bounding box localization, and significantly reduces missed detections compared to other models. These visual results confirm that YOLOv8s-BD is a more reliable solution for agricultural pest detection tasks.

To visualize the feature attention of the proposed YOLOv8s-BD, HiResCAM heatmaps are generated for pest scenes. As shown in **Figure 8**, the model accurately focuses on pest regions, with high-activation areas (yellow/red) aligning well with targets in sparse, moderately dense, and highly cluttered backgrounds. This confirms that the improved BiFPN and Dysample modules enable the network to capture discriminative features effectively, even for small or occluded pests, verifying the model’s enhanced feature representation capability.

**Figure 8:**
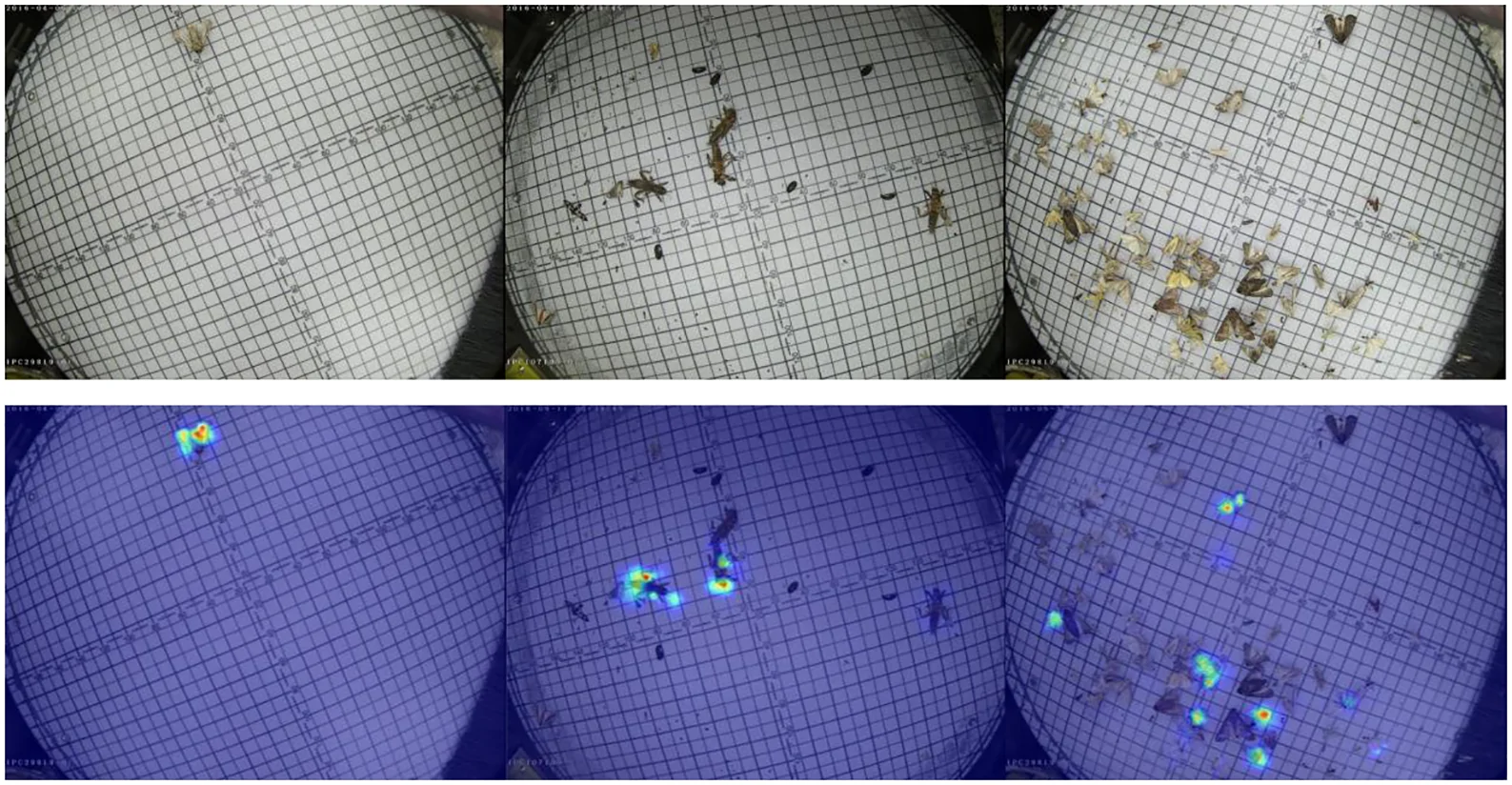
HiResCAM heatmaps for YOLOv8s-BD feature visualization.

### 3.4 Confusion matrix normalized and error analysis

To evaluate the classification performance improvement brought by YOLOv8s-BD, normalized confusion matrices of the baseline YOLOv8 and YOLOv8s-BD are presented in **Figure 9**. Each row corresponds to the true class, and each column to the predicted class, with diagonal elements representing the classification accuracy for each pest category.

**Figure 9:**
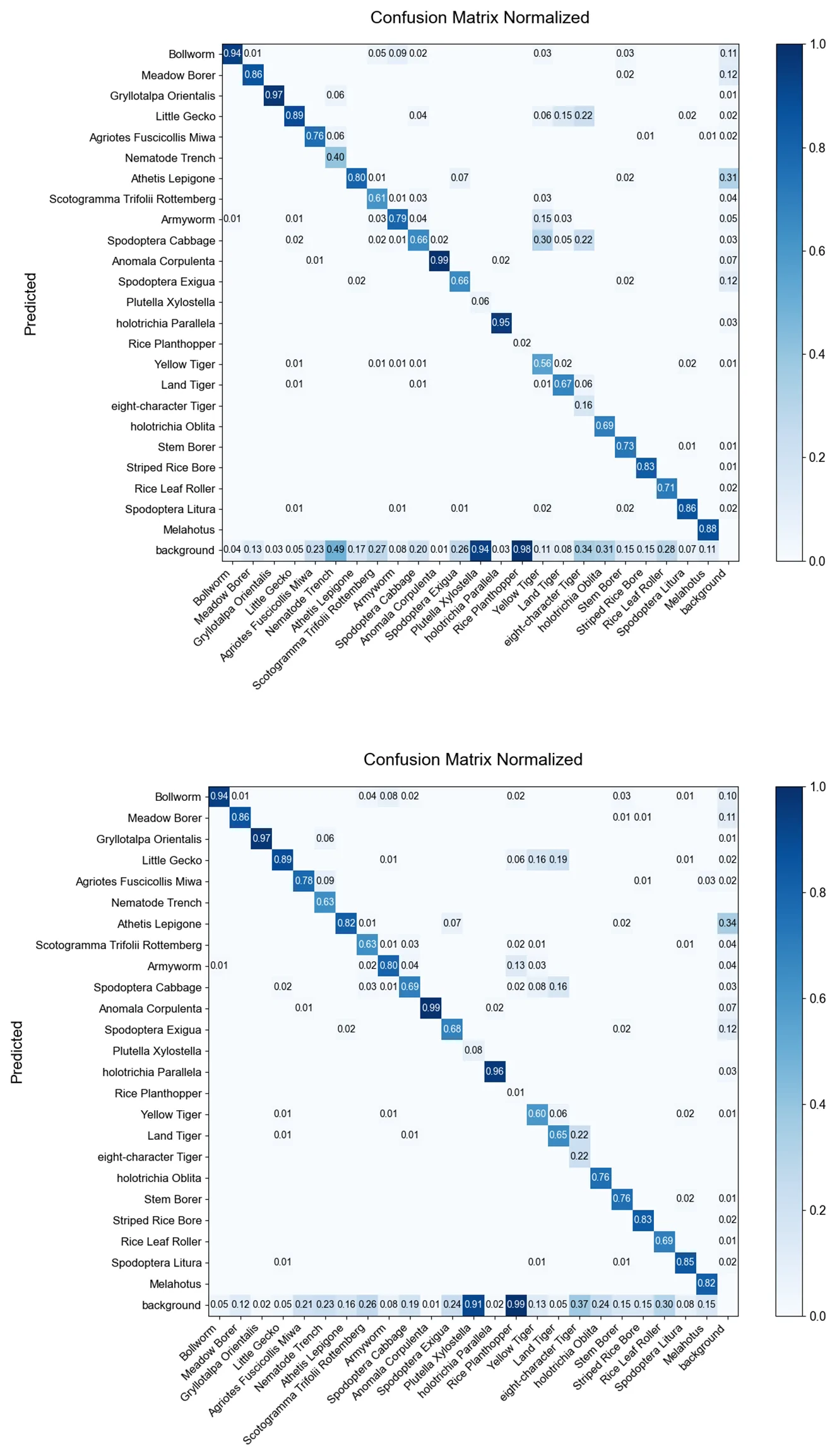
Normalized confusion matrix of YOLOv8-DB.

Compared with YOLOv8, YOLOv8s-BD achieved higher diagonal accuracy for most categories. For example, the classification accuracy of Holotrichia oblita increased from 0.69 to 0.76, while that of Melahotus changed from 0.88 to 0.82 (this slight decrease for a single category is attributed to dataset distribution, while the overall trend shows improvement for the originally lower-performing classes). More importantly, off-diagonal confusion was substantially reduced: misclassifications between morphologically similar pests (e.g., Athetis lepigone and Scotogramma trifolii Rottemberg) and between small pests and the background class were both decreased, indicating enhanced discriminative capability.

The few remaining misclassifications were concentrated in challenging cases, including small pests with low resolution and weak textural features, as well as partially occluded individuals within cluttered backgrounds. These instances continue to pose difficulties, suggesting that further enhancement of feature representation for ultra-small targets merits investigation.

Overall, the confusion matrices indicate that YOLOv8s-BD outperforms the baseline YOLOv8 in both overall classification accuracy and intra-class discriminative ability, effectively alleviating common misclassification issues in agricultural pest detection.

## 4 Discussion

The four technical barriers identified in the agricultural pest detection literature-insufficient training samples for minority classes, blurred features of tiny pests, inconsistent multi-scale feature matching, and mutual occlusion in dense pest aggregations (Liang et al., 2025; Jiang et al., 2025)-are not merely independent challenges but deeply interdependent bottlenecks that collectively degrade detection performance in real-world field settings. The Pest24 dataset adopted in this study embodies all four barriers simultaneously: it contains 24 categories of crop pests captured on transparent sticky traps under variable illumination and background conditions, with a pronounced long-tailed distribution and individual targets that routinely occupy fewer than 1% of image pixels. In such a demanding regime, designing a detection model that jointly reconciles accuracy, efficiency, and robustness requires addressing not only each barrier individually but also their mutual interactions. To this end, we designed YOLOv8s-BD as a three-component collaborative optimization pipeline comprising FastGAN-based data augmentation, DySample dynamic upsampling, and weighted BiFPN-based multi-scale feature fusion. The scientific rationale of each component and their synergistic interactions are analyzed below through the lens of ablation results and comparative experiments against the current state of the art.

A critical methodological consideration in this study is the choice of input resolution. All experiments were conducted uniformly at 640×640 pixels to ensure fair and reproducible comparisons across models. Ultra-high resolutions (e.g., 1280×1280) can partially preserve micro-textural details of sub-16-pixel pests but introduce quadratic increases in computational cost and memory footprint, making real-time edge deployment infeasible. Conversely, low resolutions (e.g., 320×320) cause irreversible loss of fine-grained features critical for distinguishing morphologically similar species-a trade-off that disproportionately penalizes rare, small-pixel categories in the Pest24 long-tailed distribution. The fixed 640×640 protocol thus represents a deliberate design choice that balances detection fidelity against practical deployability. It is important to note that existing lightweight YOLO improvement schemes for agricultural pest detection have predominantly optimized individual components in isolation-such as data augmentation pipelines (Wang P. et al., 2024), feature neck architectures (Hakim et al., 2025; Zhang et al., 2025), or attention mechanisms (Guo et al., 2025)-without addressing the mutually reinforcing coupling between feature upsampling and cross-scale fusion. This coupling constitutes the core research gap that YOLOv8s-BD is designed to bridge: poor content-agnostic upsampling degrades the spatial fidelity of high-resolution features entering the fusion pyramid, while suboptimal feature fusion fails to deliver discriminative multi-scale representations to the detection head, creating a vicious cycle that single-module improvements cannot break.

The contribution of BiFPN to detection performance can be understood through two complementary mechanisms that are particularly consequential for the Pest24 benchmark. The first is structural: by removing single-input intermediate nodes from the original PAN-FPN topology, BiFPN eliminates redundant feature transformations that introduce noise at shallow pyramid levels-levels that carry the fine-grained texture and edge cues essential for discriminating pests occupying fewer than 32×32 pixels. The second mechanism, and arguably the more impactful one for long-tailed multi-class detection, is the learnable per-level weighting. Unlike the uniform averaging in standard FPN, BiFPN assigns a distinct, gradient-optimized weight to each input feature map at every fusion node, creating an implicit attention mechanism that operates at the feature-map level. In practice, this means that high-resolution shallow features (e.g., P3, P4) carrying discriminative morphological information for small pests receive systematically higher fusion weights than deep semantic features (P5) when the target scale distribution is skewed toward small objects-as is emphatically the case for Pest24, where the majority of labeled instances occupy fewer than 1% of image pixels. The quantitative impact is substantiated by the ablation experiments: BiFPN alone increased mAP@0.5 from 70.6% to 71.8% while simultaneously reducing parameters from 11.2M to 7.4M, demonstrating that the improvement reflects targeted alleviation of shallow feature dilution rather than a generic accuracy gain from increased capacity. In contrast, YOLO-pest (Hakim et al., 2025) achieves high detection accuracy on smart trap imagery through aggressive hyperparameter tuning of YOLOv8x but retains the original PAN-FPN fusion architecture, which treats all feature levels uniformly. Similarly, Zhang et al. (2025) improved small-object detection for field crop pests through data-centric strategies while keeping the standard FPN neck unmodified. The efficiency of BiFPN-achieving higher accuracy with fewer parameters-underscores that architectural efficiency in multi-scale fusion is more decisive for pest-scale detection than model capacity alone.

DySample addresses the upsampling bottleneck through a paradigm fundamentally distinct from conventional static interpolation. Standard bilinear or nearest-neighbor upsampling applies a spatially uniform interpolation kernel regardless of the underlying object distribution, which is particularly detrimental in sticky trap scenes where multiple pests often overlap at varying depths and orientations. Under such conditions, static upsampling produces high-frequency texture aliasing at pest boundaries, causing the detector to either merge adjacent individuals into a single bounding box or generate false positive detections across background texture edges. DySample circumvents this limitation through a learnable, content-aware sampling mechanism: rather than applying a fixed interpolation formula, it generates point-wise sampling offsets conditioned on the input feature content. For a given spatial location, DySample can adaptively expand its sampling grid around pest contours while contracting it in homogeneous background regions, thereby preserving inter-pest boundary distinction even when targets are separated by only a few pixels. The ablation experiment confirms that this mechanism translates to measurable gains-mAP@0.5 improves from 70.6% to 71.3% with negligible computational overhead (parameters and GFLOPs virtually unchanged)-primarily through reduced missed detections and fewer false merges of overlapping pests. This finding aligns with recent work by Guo et al. (2025), who independently demonstrated that content-aware feature reconstruction improves small-object detection in rice disease imagery. However, unlike conditional GAN-based augmentation approaches (Wang P. et al., 2024) that generate synthetic object instances but retain standard upsampling in the detection pipeline, our joint optimization of both upsampling and fusion stages directly addresses the coupled feature degradation that single-module enhancements cannot resolve.

The synergistic gain observed when combining BiFPN and DySample-achieving 72.3% mAP@0.5 and 46.9% mAP@0.5:0.95 with only 7.4M parameters and 1.9 ms inference-reveals a fundamental insight that has not been adequately recognized in the agricultural pest detection literature: cross-scale feature fusion and spatial feature upsampling are not independent optimization targets but coupled components whose interaction can be leveraged for disproportionate gains. This 1 + 1>2 effect arises from a positive feedback loop: BiFPN’s learnable per-level weights ensure that high-resolution features receive appropriate emphasis during fusion, which in turn provides DySample with high-quality input features for content-aware upsampling; reciprocally, DySample’s adaptive reconstruction preserves fine spatial details that make BiFPN’s weighting decisions more informative and discriminative. In contrast, prior work has typically optimized these two components in isolation. For instance, YOLO-pest (Hakim et al., 2025) achieves competitive accuracy on smart trap images primarily through hyperparameter-intensive fine-tuning of YOLOv8x while retaining the original PAN-FPN architecture, and the YOLOv8-STSF detector (Wang et al., 2025) enhances multi-class pest recognition via Swin Transformer blocks but does not address the upsampling bottleneck for overlapping targets. FieldSentinel-YOLOv8 (Tan et al., 2025) employs transfer learning from Pest24 to achieve 73.64% mAP@0.5 on a rice pest dataset yet requires 10.34M parameters-39% heavier than YOLOv8s-BD-further highlighting the efficiency advantage of our jointly optimized approach. These comparisons underscore that architectural co-design, rather than modular replacement, is the more effective strategy for addressing the coupled challenges of scale variation, dense occlusion, and feature dilution that characterize real-world agricultural pest detection.

The practical implications of these findings extend beyond benchmark performance. The lightweight profile of YOLOv8s-BD (7.4M parameters, 1.9 ms per image on an A40 GPU) positions favorably for deployment on resource-constrained edge devices such as NVIDIA Jetson Orin or Raspberry Pi 5, enabling real-time pest monitoring directly in the field. For sprayer-based precision pesticide application, the end-to-end processing pipeline-comprising preprocessing (0.1 ms), inference (1.9 ms), and postprocessing (0.6 ms)-totals approximately 2.6 ms per frame, corresponding to a throughput of roughly 384 FPS. This far exceeds the 20–30 FPS acquisition rate of typical agricultural cameras. As a concrete example, for a camera with a 1 m field of view and 0.1 m detection spacing operating at 30 FPS, the system would support sprayer speeds up to approximately 10.8 km/h without any inference-induced bottleneck. Thus, the inference latency does not impose a meaningful constraint on operational sprayer speed; the primary bottlenecks lie at the camera acquisition rate and mechanical nozzle response time. Meeting these edge deployment requirements aligns with the core recommendations for intelligent plant protection systems formulated in recent comprehensive reviews of smart agriculture image recognition technology (Jiang et al., 2025).

Despite these advances, several limitations warrant careful discussion. First, the model still struggles with pests occupying fewer than 16 × 16 pixels, where residual feature information is fundamentally insufficient for reliable classification regardless of the upsampling strategy employed. This sub-pixel resolution barrier is consistent with the fundamental limitation of single-backbone lightweight architectures reported by Hakim et al. (2025), who observed that YOLO-based detectors exhibit steep performance degradation when targets fall below approximately 0.01% of the image area-a threshold that many tail-category pests in Pest24 approach. Second, minority pest precision remains suboptimal despite FastGAN augmentation. The conservative augmentation strategy adopted in this study-adding a fixed number of synthetic samples rather than forcing full class balance-preserves the natural long-tailed distribution that reflects real-world pest prevalence. However, this design choice means that rare classes (e.g., Nematode Trench, with only 767 original labels) remain at a statistical disadvantage during training. A similar accuracy gap between dominant and rare pest classes has been reported by Wang et al. (2025) in their Swin Transformer-enhanced detector, suggesting that this limitation is inherent to current long-tailed recognition paradigms rather than being specific to our architecture.

A second limitation concerns the scope of validation. The current study is confined to the Pest24 dataset, which, although comprehensive in its coverage of field crop pests, does not encompass the full diversity of agricultural imaging conditions (e.g., greenhouse environments, drone-captured imagery, or different trapping devices). Cross-dataset generalization remains a persistent challenge across the agricultural deep learning community, as systematically documented by Jiang et al. (2025), who noted that fewer than 15% of published pest detection studies include multi-dataset evaluation. Addressing this gap through domain adaptation, test-time augmentation, or the construction of unified pest detection benchmarks constitutes a priority for translating academic models into deployable field solutions.

Future work should pursue three complementary directions informed by the above analysis. First, integrating lightweight super-resolution modules or specialized attention mechanisms (e.g., deformable attention targeting sub-16-pixel regions) could extend the effective receptive field for ultra-small targets without substantially increasing inference latency, potentially pushing mAP@0.5 beyond 75% on the Pest24 benchmark. Second, few-shot or meta-learning strategies that explicitly model inter-class feature relationships could further elevate the performance of tail categories without distorting the natural class distribution-an approach that aligns with recent calls for distribution-aware training in agricultural computer vision. Third, the three-module collaborative paradigm developed here (generative augmentation + dynamic upsampling + weighted fusion) is architecturally generic and can potentially be transplanted to other fine-grained detection tasks with similar scale-imbalance challenges, such as fruit defect detection and seedling counting in precision agriculture.

## 5 Conclusion

This study proposed YOLOv8s-BD, a lightweight pest detection model designed to address the coupled challenges of tiny targets, dense distributions, high inter-class similarity, and long-tailed class imbalance in real-world agricultural scenes. By integrating FastGAN-based data augmentation to enrich training distributions, DySample dynamic upsampling to preserve fine-grained spatial details, and BiFPN-based weighted bidirectional feature fusion to optimize multi-scale representation, the model achieves 72.3% mAP@0.5 and 46.9% mAP@0.5:0.95 on the Pest24 benchmark while maintaining a lightweight architecture (7.4M parameters) and fast inference speed (1.9 ms per image). Ablation experiments confirmed a statistically significant synergistic effect between DySample and BiFPN (p<0.05), demonstrating that joint optimization of upsampling and feature fusion is substantially more effective than independent single-module improvements for multi-class tiny-pest detection. The low-latency profile and compact parameter footprint position YOLOv8s-BD as a practical candidate for real-time deployment on resource-constrained edge devices in precision agriculture. Future work will explore domain adaptation for cross-dataset generalization, few-shot learning strategies for ultra-rare pest categories, and hardware-aware model optimization for field deployment on embedded platforms.

## Data Availability

The original contributions presented in the study are included in the article/supplementary material. Further inquiries can be directed to the corresponding authors.

## References

[B1] Cen X. Lu S. Qian T. (2025). YOLO-LCE: A lightweight YOLOv8 model for agricultural pest detection. Agronomy 15, 2022. doi: 10.3390/agronomy1509202230654563

[B2] Chithambarathanu M. Jeyakumar M. K. (2023). Survey on crop pest detection using deep learning and machine learning approaches. Multimedia Tools Appl. 1500 (82), 42277–42310. doi: 10.1007/s11042-023-15221-3 37362671 PMC10088765

[B3] Gong Y. Yu X. Ding Y. Peng X. Zhao J. Han Z. (2021). Effective fusion factor in FPN for tiny object detection, in Proceedings of the IEEE/CVF Winter Conference on Applications of Computer Vision (WACV). (Piscataway, NJ: IEEE), pp. 1160–1168. doi: 10.1109/WACV48630.2021.00120

[B4] Guo L. Huang J. Wu Y. (2025). Detecting rice diseases using improved lightweight YOLOv8n. Trans. Chin. Soc. Agric. Eng. 41, 156–164. doi: 10.11975/j.issn.1002-6819.202409183

[B5] Hakim A. Srivastava A. K. Hamza A. (2025). Yolo-pest: an optimized YoloV8x for detection of small insect pests using smart traps. Sci. Rep. 15, 14029. doi: 10.1038/s41598-025-97825-3 40269001 PMC12019348

[B6] Hou S. Pang Y. Wang J. Hou J. Wang B. (2025). RS-YOLO: A highly accurate real-time detection model for small-target pest. Smart Agricultural Technology 12, 101212. doi: 10.1016/j.atech.2025.101212

[B7] Jiang C. Miao K. Hu Z. (2025). Image recognition technology in smart agriculture: a review of current applications, challenges, and future prospects. Processes 13, 1402. doi: 10.3390/pr1305140230654563

[B8] Jiang X. Chen T. Wang C. Li S. Zhang H. Zhao C. (2023). Survey of deep learning algorithms for agricultural pest detection. Trans. Chin. Soc. Agric. Eng. 39 (6), 30–44. doi: 10.3778/j.issn.1002-8331.2205-0604

[B9] Jocher G. (2020). YOLOv5 by Ultralytics (Version 7.0) [Computer software]. Zenodo, (Geneva). doi: 10.5281/zenodo.7347926

[B10] Kariyanna B. Sowjanya M. (2024). Unravelling the use of artificial intelligence in management of insect pests. Smart Agric. Technol. 8, 100517. doi: 10.1016/j.atech.2024.10051742574925

[B11] Khanam R. Hussain M. (2024). YOLOv11: An overview of the key architectural enhancements. arXiv preprint arXiv:2410.17725. doi: 10.48550/arXiv.2410.17725

[B12] Li J. Le W. Jia Z. Zhou G. Wang J. Chen G. . (2026). Small-target pest detection model based on dynamic multi-scale feature extraction and dimensionally selected feature fusion. Applied Sciences 16 (2), 793. doi: 10.3390/app16020793

[B13] Liang C. Zhu L. Zhang S. Chen J. Hou C. (2025). Research progress of crop pest detection based on machine learning. Agricultural Engineering 15 (1), 25–32. doi: 10.19998/j.cnki.2095-1795.202501304

[B14] Lin T.-Y. Dollár P. Girshick R. He K. Hariharan B. Belongie S. (2017). Feature pyramid networks for object detection, in Proceedings of the IEEE Conference on Computer Vision and Pattern Recognition (CVPR). (Piscataway, NJ: IEEE), pp. 2117–2125.

[B15] Liu W. Lu H. Fu H. Cao Z. (2023). Learning to upsample by learning to sample, in Proceedings of the IEEE/CVF International Conference on Computer Vision (ICCV). (Piscataway, NJ: IEEE), pp. 17394–17403. doi: 10.1109/ICCV51070.2023.01601

[B16] Liu S. Qi L. Qin H. Shi J. Jia J. (2018). Path aggregation network for instance segmentation, in Proceedings of the IEEE/CVF Conference on Computer Vision and Pattern Recognition (CVPR). (Piscataway, NJ: IEEE), pp. 8759–8768. doi: 10.1109/CVPR.2018.00913

[B17] Liu B. Zhu Y. Song K. Elgammal A. (2020). Towards faster and stabilized GAN training for high-fidelity few-shot image synthesis, in 8th International Conference on Learning Representations (ICLR). ( OpenReview.net).

[B18] Nie Y. Lai H. Gao G. (2025). DSOD-YOLO: A lightweight dual feature extraction method for small target detection. Digital Signal Process. 164, 105268. doi: 10.1016/j.dsp.2025.10526842574925

[B19] Palanisamy S. Gavaskar S. (2025). Enhanced pest detection in precision agriculture using YOLOv8 and EfficientNet with multi-modal fusion. International J. Appl. (Coimbatore: Bharathiar University) 38 (5).

[B20] Peng X. Zhou J. Xu Y. Xi G. (2023). Cotton top bud recognition method based on YOLOv5-CPP in complex environment. Trans. Chin. Soc. Agric. Eng. 39 (16), 191–197. doi: 10.11975/j.issn.1002-6819.202305026

[B21] Shi Y. Wang Y. Wang F. Qing S. Zhao L. Yuwen X. C. (2025). Recognizing young apples using improved YOLOv8n. Trans. Chin. Soc. Agric. Eng. 41 (8), 204–210. doi: 10.11975/j.issn.1002-6819.202408222

[B22] Sohan M. Ram T. S. Reddy C. V. R. (2024). A review on YOLOv8 and its advancements. In Data Intelligence and Cognitive Informatics. ( Springer), 529–545.

[B23] Tan S. Chen H. Zhu L. Sun H. Zhang Z. Yin L. . (2025). Identifying pests in rice fields using improved YOLOv8m. Trans. Chin. Soc. Agric. Eng. 41 (2), 185–195. doi: 10.11975/j.issn.1002-6819.202406125

[B24] Tan M. Pang R. Le Q. V. (2020). EfficientDet: Scalable and efficient object detection, in Proceedings of the IEEE/CVF Conference on Computer Vision and Pattern Recognition (CVPR). (Piscataway, NJ: IEEE), 10781–10790. doi: 10.1109/CVPR42600.2020.01080

[B25] Vilar-Andreu M. García L. Garcia-Sanchez A.-J. Asorey-Cachada R. Garcia-Haro J. (2024). Enhancing precision agriculture pest control: A generalized deep learning approach with YOLOv8-based insect detection. IEEE Access 12, 132458–132469. doi: 10.1109/ACCESS.2024.3460447

[B26] Wang C.-Y. Bochkovskiy A. Liao H.-Y. M. (2023). YOLOv7: Trainable bag-of-freebies sets new state-of-the-art for real-time object detectors. In 2023 IEEE/CVF Conference on Computer Vision and Pattern Recognition (CVPR), 7464–7473. doi: 10.1109/CVPR52729.2023.00728

[B27] Wang A. Chen H. Liu L. . (2024). Yolov10: Real-time end-to-end object detection. Adv. Neural Inf. Process. Syst. 37, 107984–108011. doi: 10.52202/079017-3429

[B28] Wang P. Ma Z. Dong B. Liu X. Ding J. Sun K. . (2024). Generative data augmentation by conditional inpainting for multi-class object detection in infrared images. Pattern Recognition 153, 110501. doi: 10.1016/j.patcog.2024.110501

[B29] Wang Q. J. Zhang S. Y. Dong S. F. Zhang G. C. Yang J. Li R. . (2020). Pest24: A large-scale very small object data set of agricultural pests for multi-target detection. Comput. Electron. Agric. 175, 105585. doi: 10.1016/j.compag.2020.105585

[B30] Wang X. W. Zhao H. N. Lu H. N. Wang Y. B. Wu D. S. Wang X. F. . (2025). Multi-category pest identification algorithm and monitoring system based on YOLOv8-STSF. Trans. Chin. Soc. Agric. Mach. 56, 228–236. doi: 10.6041/j.issn.1000-1298.2025.06.022

[B31] Yang Y. Xiao Y. Chen Z. Tang D. Li Z. Li Z. (2023). FCBTYOLO: a lightweight and high-performance fine grain detection strategy for rice pests. IEEE Access 11, 101286–101295.

[B32] Yin J. Huang P. Xiao D. Zhang B. (2024). A lightweight rice pest detection algorithm using improved attention mechanism and YOLOv8. Agriculture 14 (7), 1052. doi: 10.3390/agriculture14071052

[B33] Zhang X. Qiao H. Xi L. Ma C. Xu X. Hui X. . (2025). Detecting small objects for field crop pests using improved YOLOv8. Trans. Chin. Soc. Agric. Eng. 41 (13), 225–233. doi: 10.11975/j.issn.1002-6819.202502116

[B34] Zhen Y. Y. Liu P. Jin Y. Yang G. (2025). Grape leaf disease detection and identification based on improved YOLOv8. Trans. Chin. Soc. Agric. Eng. 41 (14), 148–154. doi: 10.11975/j.issn.1002-6819.202411033

[B35] Zheng H. Jiang Y. (2025). “ FACO-YOLO: Research on an improved underwater object detection algorithm based on YOLOv11n,” in 2025 7th international conference on information science, electrical and automation engineering (ISEAE) (Piscataway: IEEE), 784–791.

